# Exploiting heat shock protein expression to develop a non-invasive diagnostic tool for breast cancer

**DOI:** 10.1038/s41598-019-40252-y

**Published:** 2019-03-05

**Authors:** Brian T. Crouch, Jennifer Gallagher, Roujia Wang, Joy Duer, Allison Hall, Mary Scott Soo, Philip Hughes, Timothy Haystead, Nirmala Ramanujam

**Affiliations:** 1 Department of Biomedical Engineering, Duke University, Durham, NC USA; 2 Department of Surgery, Duke University Medical Center, Durham, NC USA; 3 Trinity College of Arts and Sciences, Duke University, Durham, NC USA; 4 Department of Pathology, Duke University Medical Center, Durham, NC USA; 5 Department of Radiology, Duke University Medical Center, Durham, NC USA; 6 Department of Pharmacology and Cancer Biology, Duke University Medical Center, Durham, NC USA

## Abstract

Leveraging the unique surface expression of heat shock protein 90 (Hsp90) in breast cancer provides an exciting opportunity to develop rapid diagnostic tests at the point-of-care setting. Hsp90 has previously been shown to have elevated expression levels across all breast cancer receptor subtypes. We have developed a non-destructive strategy using HS-27, a fluorescently-tethered Hsp90 inhibitor, to assay surface Hsp90 expression on intact tissue specimens and validated our approach in clinical samples from breast cancer patients across estrogen receptor positive, Her2-overexpressing, and triple negative receptor subtypes. Utilizing a pre-clinical biopsy model, we optimized three imaging parameters that may affect the specificity of HS-27 based diagnostics – time between tissue excision and staining, agent incubation time, and agent dose, and translated our strategy to clinical breast cancer samples. Findings indicated that HS-27 florescence was highest in tumor tissue, followed by benign tissue, and finally followed by mammoplasty negative control samples. Interestingly, fluorescence in tumor samples was highest in Her2+ and triple negative subtypes, and inversely correlated with the presence of tumor infiltrating lymphocytes indicating that HS-27 fluorescence increases in aggressive breast cancer phenotypes. Development of a Gaussian support vector machine classifier based on HS-27 fluorescence features resulted in a sensitivity and specificity of 82% and 100% respectively when classifying tumor and benign conditions, setting the stage for rapid and automated tissue diagnosis at the point-of-care.

## Introduction

Breast cancer management represents a complicated landscape, with therapy regimens often including a mélange of chemotherapy, radiation therapy, and surgical procedures. Unfortunately, low to middle income countries (LMICs), which shoulder most of the total breast cancer burden^1^, often do not have the resources to perform standard-of-care treatments, leading to higher mortality rates^2^. Moreover, access barriers to treatment are higher in LMICs, leading to increased time between initial medical consultation and treatment^2^. In high-income countries (HICs), when a woman presents with a suspicious lesion on her mammogram, she undergoes diagnostic biopsy to determine what type of lesion is present by pathological analysis. This strategy is not adoptable by LMICs, however, due to the scarcity of pathologists. For example, in sub-Saharan Africa the pathologist-to-population ratio is 50 times less than in HICs at approximately one to one million^3^. The distinct lack of reliable access to pathology in LMICs dictates a need for low-cost, automated methods for diagnosing breast cancer at the point-of-care. Even in HICs there are opportunities to streamline breast cancer care. For instance, in breast radiology, to ensure complete sampling of the lesion, radiologists currently take anywhere from 4–6 biopsies, which are then sent out for pathologic analysis, a process that can take up to a week. If the lesion was not successfully sampled, the patient must return for a second set of biopsies, before finally determining diagnosis and initial treatment. Similarly, in the case of Breast Conserving Surgery, evaluation of resected margins is always performed post-operatively requiring a patient to come back for re-excision if positive margins are found.

There is an opportunity for a new era of low cost, point of care molecular diagnostics to serve as an effective alternative to routine pathology. Despite its low specificity for distinguishing breast tumors from benign conditions, portable ultrasound systems are currently being used as a screening tool in lieu of mammography for breast cancer in LMICs^4,5^. A number of groups have developed methods to detect extracellular vesicles^6^ and exosomes^7,8^ extracted from blood with potential diagnostic applications for pancreatic cancer^9^ and glioblastoma^10,11^. Another example is the adaption of smart phone cameras to be used as microscopes for applications in global health^12–18^. Combining molecular diagnostics with low cost imaging technologies provides an opportunity to create low-cost, point-of-care breast cancer diagnostics for blood samples, cells, and biopsy samples.

Here, we investigated imaging Heat Shock Protein 90 (Hsp90) expression as a molecular diagnostic target in breast cancer. Hsp90 is a chaperone protein that assists other proteins to fold properly, stabilizes proteins against stress, and aids in protein degradation^19^. Hsp90 also stabilizes a number of proteins required for tumor growth^20,21^, and is overexpressed in both DCIS and invasive breast cancers^22–24^. Hsp90 is also found on the surface of many cancer types, including the breast^20,25^, and this ‘ectopic’ surface expression is specific to tumors^21^. Hsp90 inhibitors including geldanamycin analogues 17-AAG and 17-DMAG, SNX-5422 and SNX-2112, and others are currently in clinical trials^26–29^.

We have developed a fluorescently-tethered Hsp90 inhibitor, HS-27, made up of the core elements of SNX-5422, an Hsp90 inhibitor currently in clinical trials, tethered via a PEG linker to a fluorescein derivative (fluorescein isothiocyanate or FITC), that binds to ectopically expressed Hsp90, and demonstrated its potential use in a see-and-treat paradigm in breast cancer^21,30^. We found that HS-27 labels all receptor subtypes of breast cancer, but not normal cells, and specifically binds to Hsp90 expressed on the surface of breast cancer cells before being internalized. IVIS and hyperspectral imaging after systemic HS-27 injection revealed tumor selective uptake in a xenograft model, with excised tumor cryosections verifying cellular uptake. We further demonstrated that HS-27 can be used to treat aggressive Her2+ and triple negative (TNBC) breast cancers by degrading an Hsp90 client protein involved in cell metabolism, down-regulating both glycolytic and oxidative metabolism leading to decreased cell proliferation. Finally, we demonstrated an *ex vivo* imaging strategy in clinical models of breast cancer, showing all receptor subtypes of breast cancer take up HS-27 with increased fluorescence from HS-27 corresponding to areas of invasive cancer. HS-27 is a suitable candidate for use in LMICs as it does not require refrigeration and can be made inexpensively when made to scale.

In this study we focused on optimizing imaging parameters including post-excision window, incubation time, and agent dose to rapidly translate HS-27 to clinical use by excising murine breast tumors (4T1) and staining them *ex vivo*. With optimized imaging parameters of a 1 to 10-minute post-excision window, 1-minute incubation time, and 100 µM dose, we then demonstrated the feasibility of our imaging strategy on standard of care biopsies from patients presenting with a mammographic lesion, as well as a population of patients undergoing breast reduction mammoplasty to interrogate HS-27 uptake by normal breast tissue. To determine potential sources of HS-27 fluorescence, we investigated correlations between HS-27 fluorescence and the density of cancer or tumor stromal cells to assess whether density of tumor cells and surface Hsp90 expression dictate fluorescence levels. We further examined correlations between HS-27 fluorescence and the density of tumor infiltrating lymphocytes (TILs), a positive prognostic marker in breast cancer, as well as breast cancer receptor subtypes to investigate whether or not surface Hsp90 is further up-regulated by aggressive tumors. Finally, we employed image processing methods to extract HS-27 fluorescence features to differentiate tumor from benign tissues.

## Results

### Optimization of HS-27 incubation parameters for *ex vivo* imaging

We optimized three distinct imaging parameters in preclinical studies that could potentially affect the specificity of HS-27 uptake by clinical samples. The first parameter we investigated was the time between excision and staining (1, 3, or 10 minutes) to understand how ectopic Hsp90 expression changes as time between excision and application of the contrast agent is increased. The second parameter was HS-27 incubation time (1, 5, or 10 minutes), which when increased may increase non-specific HS-27 diffusion into the tissue. Finally, we optimized agent dose (1, 10, 50, or 100 µM) to round out our investigation. For optimization, the specificity of HS-27 uptake was defined by the ratio of HS-27 (specific signal) to HS-217 (non-specific HS-27 analog signal) fluorescence.

**Figure 1:**
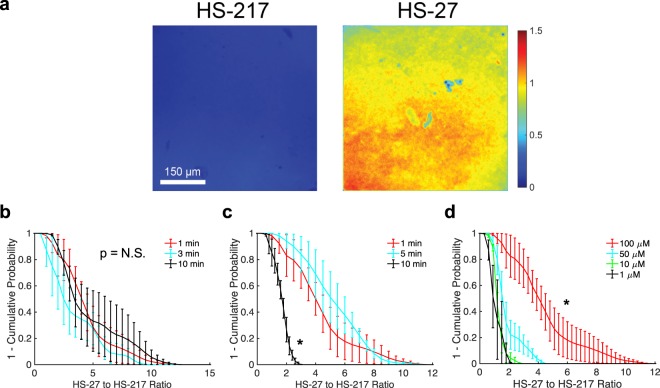
A 1 to 10-minute post-excision window, 1-minute incubation time, and 100 µM dose maximizes the HS-27 to HS-217 specificity ratio. 4T1 tumors were biopsied and incubated in either 100 µM HS-27 or 100 µM HS-217 for 1-minute either 1-minute, 3-minutes, or 10-minutes post biopsy prior to fluorescence imaging to identify the optimal post-excision window, or 1-minute post excision for 1-minute, 5-minutes, or 10-minutes to identify the optimal agent incubation time. To identify optimal dose, biopsies were incubated in either 1 µM, 10 µM, 50 µM, or 100 µM HS-27 or HS-217 1-minute post-excision for 1-minute prior to fluorescence imaging. (**a**) Representative fluorescence images of 4T1 biopsies stained with 100 µM HS-217 or HS-27 for 1-minute within 1-minute of tissue excision. (**b**–**d**) Survival curves of the ratio of HS-27 to HS-217 fluorescence demonstrate no significant differences with increasing post-excision time (**b**), a significant decrease in the specificity ratio with increasing incubation time (**c**), and a significant increase in specificity ratio with increasing dose (**d**) by Kolmogorov-Smirnov (KS) test. For all groups n = 4 biopsies. Survival curves show the mean ± SEM.

Representative fluorescence images of HS-27 or HS-217 biopsies from 4T1 murine breast tumors treated with the optimized parameters, shown in Fig. 1a, demonstrate that HS-27 signal is significantly greater than non-specific HS-217 signal. Representative images from post-excision window, incubation time, and dose experiments can be found in Supplementary Fig. S1. Curves of HS-27 to HS-217 fluorescence ratio fractions (survival curves defined as 1 minus the cumulative probability), clearly indicate the optimal imaging parameters (Fig. 1b–d). The ratio of HS-27 to HS-217 signal showed no significant changes when increasing the post-excision window from 1 to 10 minutes, as shown in Fig. 1b. Conversely, Fig. 1c shows that increasing agent incubation time from 1 or 5-minutes to 10-minutes significantly decreased specificity. Finally, 100 µM agent dose showed the greatest specificity in Fig. 1d. For all groups n = 4 biopsies. A 1-minute post-tissue excision time, 1-minute incubation, and a 100 µM dose were established as the parameters to use in the clinical studies.

### HS-27 fluorescence is greater in tumor than non-tumor tissue

**Figure 2:**
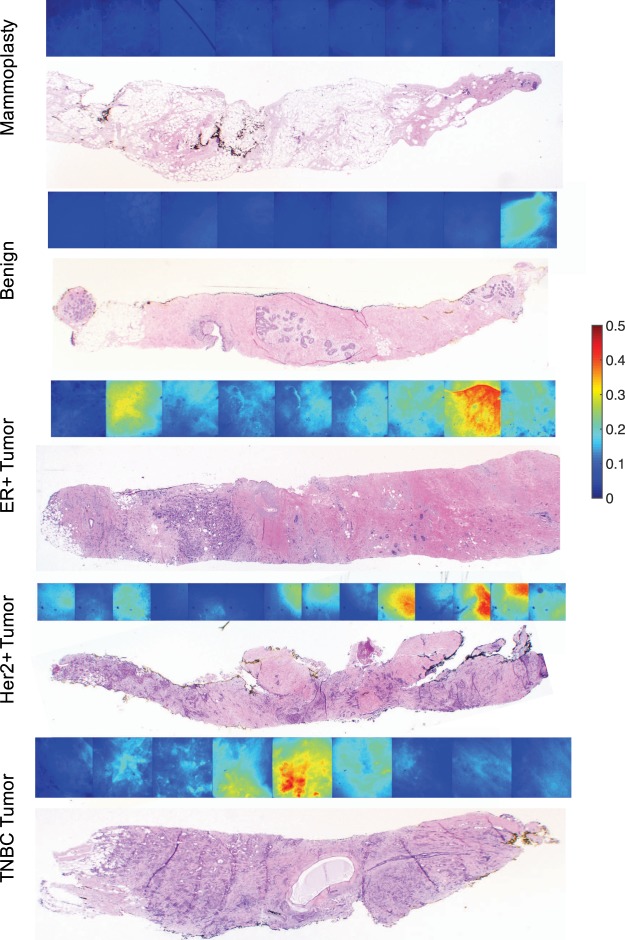
HS-27 uptake is greater in tumor than non-tumor samples. USGCNB were obtained from patients prior to imaging with our optimized parameters. Representative fluorescence (top) and histology (bottom) images of mammoplasty tissue, fibroadenoma, ER/PR-positive tumor, TNBC, and Her2-positive tumor.

Next, the protocol we established in pre-clinical studies was applied to biopsies obtained from patients undergoing ultrasound guided core needle biopsy (USGCNB). Typically the first biopsy from each patient was imaged to increase the likelihood of obtaining biopsies with cancer. Images were obtained in 1 mm increments along the biopsy prior to inking to ensure proper orientation for site-level pathology, as previously described^30^. Representative biopsy images from an ER/PR-positive tumor, Her2-overexpressing tumor, TNBC, benign lesion (fibroadenoma), and normal mammoplasty tissue demonstrate greater HS-27 fluorescence in tumor compared to benign and normal tissues as shown in Fig. 2. Histology H&E images from the sites that were imaged are shown below for comparison.

**Figure 3:**
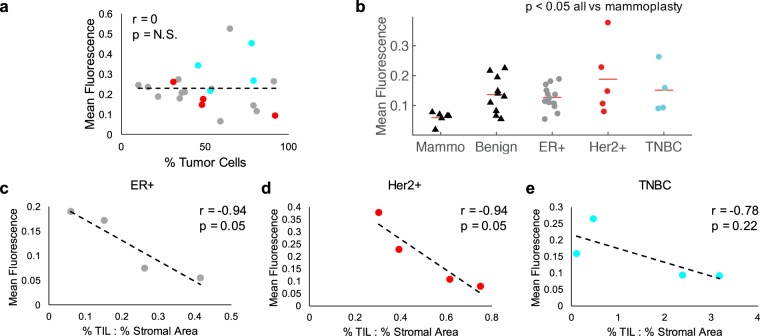
Receptor subtype and presence of TILs affect HS-27 fluorescence levels more than tumor cellularity. (**a**) HS-27 fluorescence does not correlate with tumor cellularity. (**b**) Mean fluorescence varies with receptor subtype and is significantly lower in mammoplasty than all other tissue types. (**c**–**e**) Mean fluorescence strongly and inversely correlates with the density of TILs across receptor subtypes.

We next wanted to understand the potential sources of HS-27 fluorescence within a biopsy image. There are three potential subsets of cell types present within a malignant biopsy – cancer cells, tumor associated stromal cells, and surrounding benign cells. Our pathologist assessed each 1-mm site along the biopsy for percent tumor area (PTA), tumor cellularity (the percentage of the tumor area made up of cancer cells), and stromal area (1-tumor cellularity). The density of tumor infiltrating lymphocytes (TILs) within the stromal area was also provided. We began by investigating the relationship between mean HS-27 fluorescence and tumor cellularity and found that there was no correlation between the two endpoints, as shown in Fig. 3a. Since tumor cellularity did not correlate with HS-27 fluorescence, we next examined how mean HS-27 fluorescence varied with receptor subtype, as shown in Fig. 3b. HS-27 fluorescence was highest in Her2+ tumors, followed by TNBC, and ER+. Next, we looked at how the presence of various tissue types influenced fluorescence. Based on our previous study suggesting surface Hsp90 is upregulated in particularly aggressive tumors^30^, we explored the relationship between HS-27 fluorescence and the percent of tumor infiltrating lymphocytes (TILs), a positive prognostic factor in Her2+ and TNBC receptor subtypes^31–33^. Because TILs are given as a percentage of stromal area covered by TILs, we took the ratio of TIL% to stromal % to provide a more accurate density of TILs in the biopsy. HS-27 fluorescence is inversely correlated with increased density of TILs in the tumor stroma across receptor subtypes, as shown in Fig. 3c–e. These results suggest that receptor subtype and the density of TILs more strongly influences the mean fluorescence than tumor cellularity.

### HS-27 fluorescence features accurately distinguish tumor from benign tissue

**Figure 4:**
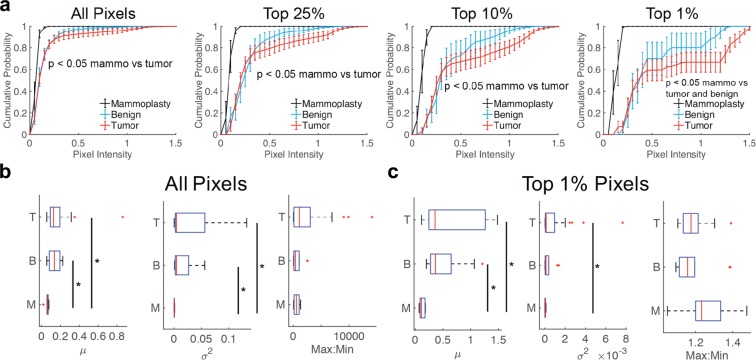
HS-27 fluorescence is greater in tumor than non-tumor tissue. (**a**) CDFs of fluorescence image pixel intensities were created for each combined biopsy image of either all pixels or of only the top 25%, top 10%, or top 1% of pixels. Curves were stratified by histology type. Mammoplasty (black) survival curves were significantly different from tumor curves by KS testing across pixel bins. (**b**,**c**) Box plots of intensity summary parameters mean, variance and max to min ratio for tumor (T), benign (**b**) and mammoplasty (M) biopsies for (**b**) all pixels or (**c**) the top 1% of pixels. Sample sizes – n = 6 mammoplasty, n = 10 benign, n = 27 tumor. *p < 0.05 by KS testing (CDFs) or one-way ANOVA with Tukey-Kramer post-hoc testing (box plots). Survival curves show the mean ± SEM.

One of the major challenges of traditional mammography is the ability to distinguish benign from malignant conditions, hence the need for biopsies and subsequent histopathology. We wanted to examine whether or not features from HS-27 fluorescence images could be used to distinguish benign from malignant tissues and serve as a potential alternative for histopathology. Fig. 4a shows cumulative distributions (CDFs) of HS-27 fluorescence intensity from the full stitched image for tumor vs. benign vs. mammoplasty tissue. Because many of our lesion images contain non-lesion regions, we utilized distributions to test cut-off thresholds to include all pixels or only the top 25%, top 10%, or top 1% of pixels to increase the specificity of HS-27 based diagnostics. Clearly, for the top 1% of pixels, there is an increase in separation between the curves, reflected by decreasing p-values determined from Kolmogorov-Smirnov (KS) testing. Though not significant, tumor fluorescence is greater than benign across all bins, and significantly different than mammoplasty control tissues across all bins. Benign is only significantly different from mammoplasty at the 1% pixel bin level.

We created 12 different parameters from our fluorescence images that could be used as optical predictors to distinguish tumor from both benign lesions and normal breast tissue from mammoplasty cases. The first 6 were calculated by fitting a logistic curve to each CDF from either all pixels or the top 1% of pixels with summary variables *A*, *B*, and *C*, as shown in Supplementary Fig. S2. Parameter *A* controls the slope of the CDF, reflecting primarily the variance of pixel values within each image. Parameter *B* controls the left/right shift of the CDF, reflecting primarily the mean pixel value within each image. Parameter *C* controls the vertical shift of the CDF, reflecting both the mean and variance of the highest pixel values. The remaining 6 parameters were calculated as summary parameters, namely the overall mean, variance, and ratio of the maximum to minimum fluorescence for all pixels and the top 1% of pixels to report on the average fluorescence, fluorescence spread, and dynamic range respectively. Boxplots of the summary variables across all pixels and the top 1% of pixels are shown in Fig. 4b,c respectively.

We next explored how the CDF and summary parameters affect the accuracy of HS-27 based classification. Since there are differences (though not all significant) between tumor, benign lesion, and mammoplasty tissue types, we performed two sets of comparisons – tumor vs. mammoplasty and tumor vs. benign lesion. For the two comparison groups, Gaussian support vector machine (GSVM) classifiers were created and tested with 10-fold cross-validation to create receiver operating characteristic curves (ROCs) using either the CDF fit parameters for all pixels, the CDF fit parameters for the top 1% of pixels, the summary variables for all pixels, or the summary variables for the top 1% of pixels. The sensitivity, specificity, and area under the curve (AUC) for the ROC for each scenario are summarized in Table 1.

**Table 1:** Summary of GSVM performance for tumor vs mammoplasty (T v. M) and tumor vs benign (T v. B) classifiers.

Comparison	CDF_All_	CDF_Top 1%_	Summary_All_	Summary_Top 1%_	Sens.	Spec.
T v. M	0.8				52%	100%
T v. M		0.8			93%	67%
T v. M			0.95		89%	100%
T v. M				0.85	78%	100%
T v. B	0.78				67%	90%
T v. B		0.74			74%	70%
T v. B			0.44		93%	20%
T v. B				0.72	44%	100%

The AUC is shown in the box corresponding to the parameters used for classifier development.

Looking at the AUCs in Table 1 reveals an interesting pattern. For tumor vs. mammoplasty comparisons, utilizing the CDF fit parameters from the top 1% of variables achieved a higher AUC than utilizing the CDF fit parameters from all pixels. The converse was true for tumor vs benign lesion comparisons. Similarly, utilizing the summary parameters for all pixels achieved a higher AUC than the summary parameters from the top 1% of pixels for tumor vs mammoplasty samples, with the opposite holding true for tumor vs benign comparisons.

**Figure 5:**
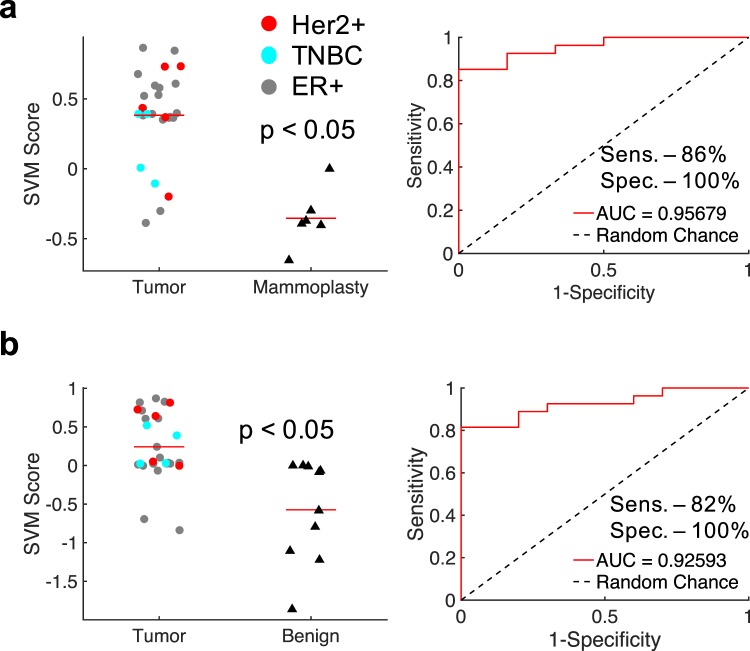
HS-27 features distinguish tumor from both mammoplasty and benign tissues. Gaussian support vector machine (GSVM) classifiers were developed for combinations of CDF and summary variables for both tumor vs mammoplasty and tumor vs benign tissues. (**a**) GSVM scores and an ROC for a GSVM classifier for distinguishing tumor from mammoplasty tissue based on the variance of all pixels and the CDF C parameter from the top 1% of pixels. (**b**) GSVM scores and an ROC for a GSVM classifier for distinguishing tumor from benign tissue based on the variance of the top 1% of pixels and the CDF C parameter from all pixels.

We performed a sequential feature selection method to identify the optimal 2 parameters for tumor vs mammoplasty and tumor vs benign lesion comparisons, by testing all combinations of the 12 parameters using a GSVM with 10-fold cross-validation. The optimal parameters were chosen as those that led to the highest AUC for the corresponding ROC. A combination of a summary parameter from all pixels (variance) and a CDF parameter from the top 1% of pixels (CDF C) performed the best for tumor vs mammoplasty comparisons. GSVM scores and the ROC for the optimal tumor vs mammoplasty GSVM are shown in Fig. 5a. The optimal sensitivity and specificity were determined by maximizing the Youden’s index, and were 86% and 100% respectively, with an AUC of 0.96. In line with the results from Table 1, the same variables in opposite pixel bins performed best for tumor vs benign lesion comparisons (variance of the top 1% of pixels and the CDF C parameter for all pixels). GSVM scores and the ROC for the optimal tumor vs benign GSVM are shown in Fig. 5b. The optimal sensitivity and specificity were again determined by maximizing the Youden’s index, and were 82% and 100% respectively, with an AUC of 0.93.

## Discussion

With the complexity of breast cancer care continually increasing, and the associated cost burdens mounting, it is more important to streamline care now more than ever before. When establishing our *ex vivo* diagnostic methodology, simplicity and cost were two major considerations. We have created an *ex vivo* imaging strategy to image surface Hsp90 expression in breast tumor biopsies. We optimized HS-27 uptake in pre-clinical models, and found that a post-excision window of 1 to 10-minutes, incubation time of 1-minute, and dose of 100 µM resulted in the greatest specificity ratio. Translating this protocol to clinical biopsy samples, we demonstrated significantly greater HS-27 uptake in tumor vs mammoplasty control tissues, and found that both cancerous and tumor stromal cells contribute to HS-27 fluorescence. GSVM analysis achieved an AUC of 0.93 with a sensitivity and specificity of 82% and 100% respectively.

Interestingly, we found that benign breast conditions like fibroadenoma, abnormal ductal hyperplasia, and cystic tissue showed higher HS-27 fluorescence than mammoplasty control tissue, suggesting the presence of surface Hsp90 in these samples, though at a lower level than in tumors. It is possible there is some surface Hsp90 expression in benign samples as indicated by HS-27 fluorescence signal. Surface Hsp90 in benign samples may be a mechanism of immune cell recruitment, as there have been numerous studies demonstrating the role Hsp90 plays during immune responses, both innate and adaptive^34–37^. For example, in innate immunity the presence of Hsp90 extracellularly can signal a damage associated molecular pattern causing immune cell recruitment^34^. The induction of surface Hsp90 expression to activate immune responses during benign conditions reduces the sensitivity for identifying tumor lesions. That being said, we still found using a non-linear GSVM using both intensity and spatial HS-27 fluorescence based predictors yielded the highest sensitivity and specificity.

Our algorithm incorrectly classified 5 tumor biopsies as benign lesions. All of these biopsies came from women with ER+ breast cancer, with one biopsy also showing over-expression of Her2. In our previous pre-clinical studies, we have found that Her2+ and TNBC have greater surface Hsp90 expression that ER+ tumors^30^. Even so, we still correctly classified 71% of our ER+ tumors. Deep learning techniques may be better suited to address this limitation of our approach, however, due to the small sample size of this study, we are limited in the machine learning techniques we can apply to our dataset. It is also important to note that the small sample size dictates further larger scale studies to validate these results. In the future, we plan to develop more advanced deep-learning non-linear strategies, like artificial neural networks, to improve the overall performance of our diagnostic platform. If there is insufficient contrast between ER+ tumors and benign tissues, due to the relatively low expression of Hsp90 in these tumors, a combination of contrast agents may be used to enhance sensitivity extending the capabilities of our platform.

We noticed some heterogeneity in uptake both within and across biopsies. Each biopsy is comprised of many different cell types that may have varying levels of surface Hsp90 expression (i.e. malignant cells, tumor associated fibroblasts, tumor infiltrating lymphocytes, and non-malignant cells such as adipocytes), which would influence HS-27 uptake and may cause some of the intra-biopsy heterogeneity in HS-27 fluorescence. This is further evidenced by the considerably greater homogeneity seen in the mammoplasty images, which are primarily adipocytes.

In our clinical study we found variable HS-27 uptake within receptor subtypes, which, when coupled with the established relationships between Hsp90 and immune responses, potentially provides an endpoint useful for guiding treatment. For example, surface Hsp90 expression may be a useful surrogate marker for tumor infiltrating lymphocytes (TILs), which are of particular importance, as increased density of TILs in patients with early stage Her2+ breast cancer showed increased pathological complete response (pCR) when treated with standard-of-care therapies trastuzumab and/or lapatinib^32,33^. Further, increased levels in TNBC have been associated with improved patient outcomes following treatment with anthracycline-based chemotherapies^31^. Interestingly, when binning Her2+ and TNBC samples together, we found a strong and significant inverse correlation between HS-27 fluorescence and the density of stromal TILs (r = −0.63, p < 0.05). Despite promising retrospective studies demonstrating the prognostic significance of TILs, there are some limitations to using TIL involvement as a prognostic or predictive biomarker in a clinical setting. Although efforts have been made to standardize the assessment of TILs^38^, this assessment is still subject to inter-observer variability. The evidence of Hsp90 involvement in immune regulation combined with our own findings in Her2+ and TNBC tumors provides a compelling opportunity to explore how surface Hsp90 expression on carcinoma cells relates to the immune cell milieu in the tumor microenvironment.

Other groups are exploring molecular imaging techniques for applications in cancer^39–41^, including a group performing *ex vivo* imaging of breast tumors for applications in margin assessment using Her2-targeted fluorescent antibodies^42^. Utilizing a dual-probe approach with targeted and non-targeted antibodies at different fluorescence wavelengths allowed for highly accurate identification of tumors vs non-tumor tissue. Similar to the optimization results in our study, they found that shorter incubation times yielded increased imaging specificity. Other groups are utilizing quantum dots tethered to antibodies^43^ to identify protein biomarkers such as the estrogen receptor as well as more ubiquitously expressed targets like EGFR^44,45^ for diagnostic purposes in estrogen receptor positive patients. Our work builds on tumor-specific imaging by targeting surface Hsp90 expression, which is ubiquitous to all receptor subtypes of cancer, increasing the potential population target from only Her2+ tumors (~20% of breast cancer diagnoses) to all patients with breast cancer. Additionally, by utilizing a small molecule specific to Hsp90 rather than antibodies, our approach does not require any initial blocking steps to prevent non-specific binding, reducing the required processing to tissue and imaging time. Finally, by reducing the cost of both the molecular agent and imaging system, we are primed to provide rapid diagnostic information to physicians even in settings where on-site pathology is not possible.

In high-income countries (HICs), tumor specific targeting with HS-27 will allow for rapid analysis of biopsies during diagnostic biopsy and tumor margins in the OR. A careful examination of each tissue type (tumor, benign lesion, mammoplasty) reveals different HS-27 uptake patterns necessitating different metrics to separate tumor from benign lesions and tumor from healthy (mammoplasty) tissue. In the biopsy clinic, the possible tissue types are either tumor or benign lesion, dictating use of the GSVM algorithm based on tumor vs. benign samples. For margin assessment, the possible tissue types are either tumor or healthy tissue, dictating use of the GSVM algorithm based on tumor vs. mammoplasty samples.

Performing our imaging *ex vivo* circumvents the need for the regulatory approvals required for *in vivo* applications in fluorescence guided surgery, and decreases the risk of side effects to the patient. In our model, the primary tumor (or biopsy) will be rapidly assayed for the presence of disease, finding the equivalent of pathological tumor on ink, normally necessitating a re-excision. Tumor cells will be selectively visualized using HS-27, and localized by easily navigating back and forth between wide-field and high-resolution imaging with our Pocket mammoscope, a fluorescence microscope adapted from our widely-used Pocket colposcope^46–48^. When disease is found on the margin surface, the surgeon will go back and take additional shavings from the surgical cavity. This strategy will be repeated until there is no signal on the surface of the margins.

Fortuitously, the ability to image tumor immune responses may fill an important niche in cancer prognostics as well. Currently, for neoadjuvant and adjuvant treatment decisions oncologists use a combination of clinical factors determined from either a diagnostic biopsy (neoadjuvant) or surgical specimen (adjuvant)^49,50^. Unfortunately, current clinical factors such as hormone and/or growth factor receptor status are insufficient to predict which patients are likely to benefit from additional therapies^51^. Without predictive tests, patients may receive unnecessary and/or ineffective treatments, which increases costs on an already overburdened healthcare system, and exposes patients to unnecessary toxic side effects. Genetic tests including Oncotype DX have been developed to assess whether ER+ breast cancer patients are likely to benefit from adjuvant chemotherapy, reducing the use of potentially toxic therapies for women with low recurrence risk. However, for the 30% of patients with either Her2+ or triple negative breast cancer (TNBC), there is no well-established predictive test to guide treatment, beyond standard hormone receptor and Her2 testing. Being able to use Hsp90 expression as a surrogate for TIL levels may allow for a way to extend the prognostication available to patients with ER+ breast tumors to patients with HER2+ or triple negative breast cancers.

There is often significant patient attrition when multiple visits are required to diagnose and treat breast cancer in LMICs. Integrating diagnostics with an effective treatment strategy into a single visit will improve outcomes for patients in LMICs where standard of care pathology and surgical treatments are not feasible. The combination of HS-27 with a low-cost microscopy system will provide a cost-effective and easily implementable diagnostic platform for breast cancer as a first step towards a single-visit see-and-treat strategy.

## Methods

### Cell culture

4T1 murine breast cancer lines were used in the pre-clinical study, and were acquired from the American Type Culture Collection and cultured under standard conditions free of contamination at 37 °C and 5% CO_2_. Cells were maintained in RPMI-1640 (L-glutamine) medium supplemented with 10% FBS and 1% penicillin-streptomycin. All cells were used for experiments within one month of first passage.

### Animal studies

All animal experiments were performed in accordance with protocol A216-15-08 approved by the Duke University Institution for Animal Care and Use Committee. Animals were housed on-site with continual access to food and water under normal 12-hour light/dark cycles.

### Flank tumor biopsy model

4T1 tumors were grown in the flank of 11 athymic nude mice for optimizing *ex vivo* imaging parameters. Specifically, on passage two after thaw, 10^6^ 4T1 cells suspended in 100 µL serum-free medium were injected subcutaneously into the right flank to establish tumors. Tumors were allowed to grow to a volume of 1 cm^3^ (tumor volume calculated as 0.5 × length × width^2^) to form a mass similar in size to those evaluated in clinical radiology. We have previously described our biopsy procedure in detail^30^. Briefly, mice were anesthetized with a maximum of 1.5% isoflurane in room air. Prior to biopsy, scissors were used to make a small incision to remove the skin over the tumor. Biopsies were taken using a 12 gauge Achieve programmable automated biopsy system. Three biopsies were taken from random locations within each tumor for 10 mice, with two biopsies taken from the remaining mouse, yielding 32 biopsies for analysis.

### Pre-clinical *ex vivo* imaging optimization

We identified and sequentially optimized three parameters that could affect the specificity of HS-27 uptake: (1) time between tissue excision and staining, (2) agent incubation time, and (3) agent dose. Because HS-27 is a small molecule rather than an antibody, no blocking steps or specialized washes are required prior to agent incubation. For parameter 1, time between tissue excision and staining was varied from 1, to 3, to 10-minutes while agent incubation time and dose were fixed at 1-minute and 100 µM respectively. For parameter 2, incubation time was varied from 1, to 5, to 10-minutes while time after tissue excision and agent dose were fixed at 1-minute and 100 µM respectively. Finally, for parameter 3, dose was increased from 1, to 10, to 50, to 100 µM while time post excision and agent incubation time were both fixed at 1-minute. 8 biopsies were used for each group in each experiment with 4 biopsies receiving HS-27 treatment and 4 biopsies receiving HS-217 treatment. HS-217 is a non-specific version of HS-27 that does not bind to Hsp90 and serves as a negative control^30^. After HS-27 or HS-217 incubation, biopsies were thoroughly rinsed once with PBS to remove unbound probe. Images were collected using a high-resolution microendoscope (HRME)^52^ every mm along the length of the biopsy and stitched together for analysis as previously described^30,53^.

### Clinical *ex vivo* biopsy imaging

All clinical imaging was performed in accordance with Duke IRB approved protocol number Pro00008003. After giving informed consent, 34 adult patients undergoing standard of care ultrasound guided core needle biopsy (USGCNB) and 4 adult patients undergoing breast reduction mammoplasty were enrolled in our study. Breast reduction mammoplasty patients serve as a negative control. Of the 34 USGCNB patients, one biopsy was imaged from each patient except in three patients where two biopsies were imaged: one from each of two masses. Of the 4 reduction patients, two biopsies were imaged from each of two patients and one from each of the remaining patients, resulting in 37 USGCNBs and 6 mammoplasty biopsies for analysis. Of the 37 USGCNBs, 27 were invasive ductal cancer, of which 17 were ER/PR-positive, 4 were ER/Her2-positive, 1 was ER/PR-negative but Her2-positive, and 4 were triple negative. The remaining 10 were benign conditions and all mammoplasty samples were normal breast tissues. Table 2 summarizes the demographic and histologic information from our patient population.

**Table 2:** Demographic breakdown of patients enrolled in pilot clinical study.

Characteristic	Biopsies
***Number of Patients***	
Ultrasound (US) biopsy	37
Mammoplasty biopsy	6
***Patient Demographics***	
Average Age (range)	55 (25–79)
Average BMI (range)	30 (18–54.4)
***Pathology Breakdown***	
Malignant (US only)	27 (73%)
Benign (US only)	10 (27%)
***Receptor Status (malignant only)***	
ER+,−	22 (81%), 5 (19%)
PR+,−	19 (70%), 8 (30%)
Her2+,−	5 (19%), 22 (81%)
TNBC	4 (15%)
**Menopausal Status**	
Pre-menopause	14 (37%)
Peri-menopause	1 (2%)
Post-menopause	23 (61%)
***Breast Density***	
Fatty	4 (11%)
Scattered Fibroglandular	13 (35%)
Heterogeneous Density	15 (41%)
Extremely Dense	5 (13%)

Each biopsy was received within 5-minutes of tissue excision and had 100 µM HS-27 topically applied to the biopsy for 1-minute prior to thorough rinsing with PBS. Images were collected using the HRME every mm along the length of the biopsy and stitched together for analysis as previously described^30,53^. Biopsies were then inked in three different colors and sent for standard pathologic review by a trained pathologist. The pathologist (AH) provided specific diagnoses every mm along the biopsy for co-registration with HRME images, including the percent tumor area (PTA), tumor cellularity, and the percent benign tissue area.

### HS-27 fluorescence quantification

All HRME images were processed using MATLAB (MathWorks). Both pre-clinical and clinical images were calibrated using a fluorescence slide to account for day-to-day system variations. Because the HRME camera uses an automatic gain and exposure time, HRME images were post-processed to correct for differences between imaging sessions. For pre-clinical HS-27 uptake optimization, non-specific fluorescence was assessed by calculating the mean pixel intensity from the HS-217 images corresponding to each optimization parameter and variable. The specificity ratio was then calculated by dividing each HS-27 image by the corresponding mean HS-217 fluorescence. Cumulative pixel distributions (CDFs) for each image were averaged across biopsies within a group and used for statistical comparison.

### Image processing, feature extraction and selection, and Gaussian support vector machine classification

The 37 USGCNB images were binned into either tumor (n = 27) or benign (n = 10) groups based on their pathological diagnosis. 12 different parameters were created from our fluorescence images to be used as optical predictors. 6 were calculated as the mean pixel value, variance of pixel values, and maximum to minimum pixel value ratio from either all or the top 1% of pixels. To take advantage of the intensity distributions within the images, a logistic curve was fit to the cumulative distribution function (CDF) to either all pixels or the top 1% of pixels for each biopsy with three parameters per CDF that represent the CDF slope (*A*), the left/right shift (*B*), and the vertical shift of the top of the CDF (*C*), as shown in Supplementary Fig. S2.

Next, we created Gaussian support vector machine (GSVM) classifiers using the MATLAB Machine Learning Toolbox to distinguish tumor from mammoplasty samples and tumor from benign lesion samples. A sequential feature selection method was used to select the optimal set of features for each classifier by testing each feature individually, and then all possible pairs of features. The feature(s) resulting in optimal separation of tumor and benign samples then underwent 10-fold cross validation. A receiver operating characteristic (ROC) curve was generated for the optimized GSVM classifier, and the optimal sensitivity and specificity were determined by maximizing the Youden’s index.

### Statistical analysis

A two-sided student’s t-test was used for experiments comparing only two groups. A one-way ANOVA with Tukey-Kramer post-hoc testing was used for experiments comparing more than two groups. CDFs were compared using a Kolmogorov Smirnov (KS) test. Pearson’s linear correlations were used to calculate correlation coefficients. Comparisons and correlations were considered significant on a 95% confidence interval with a p-value of 0.05 or less. All statistical testing was performed using the Statistics Toolbox in MATLAB (MathWorks).

## Supplementary information

Supplementary Figures

## Data Availability

The datasets generated during and/or analyzed during the current study are available from the corresponding author on reasonable request.
