# Supplementary material for: Exploiting heat shock protein expression to develop a non-invasive diagnostic tool for breast cancer
Source: Sci Rep. 2019 Mar 5;9:3461. doi: 10.1038/s41598-019-40252-y (PMC6400939; doi:10.1038/s41598-019-40252-y)
Supplement: Supplementary file 1 — Supplementary Figures [file 41598_2019_40252_MOESM1_ESM.pdf]

Title: Exploiting heat shock protein expression to develop a non-invasive diagnostic tool for breast cancer

Authors

Brian T. Crouch Ph.D.[1*], Jennifer Gallagher[2], Roujia Wang[1], Joy Duer[3], Allison Hall M.D. Ph.D.[4], Mary Scott Soo M.D.[5], Philip Hughes Ph.D.[6], Timothy Haystead Ph.D.[6], Nirmala Ramanujam Ph.D.[1,6]

Affiliations

[1] Department of Biomedical Engineering, Duke University, Durham, NC, USA

[2] Department of Surgery, Duke University Medical Center, Durham, NC, USA

[3] Trinity College of Arts and Sciences, Duke University, Durham, NC, USA

[4] Department of Pathology, Duke University Medical Center, Durham, NC, USA

[5] Department of Radiology, Duke University Medical Center, Durham, NC, USA

[6] Department of Pharmacology and Cancer Biology, Duke University Medical Center, Durham, NC, USA

Contact Information

*Department of Biomedical Engineering

1427 FCIEMAS, 101 Science Dr.

Campus Box 90281

Durham, NC 27708-0281

Phone: 919-660-8473

Fax: 919-684-4488

Email: brian.crouch@duke.edu

Supplementary Figures

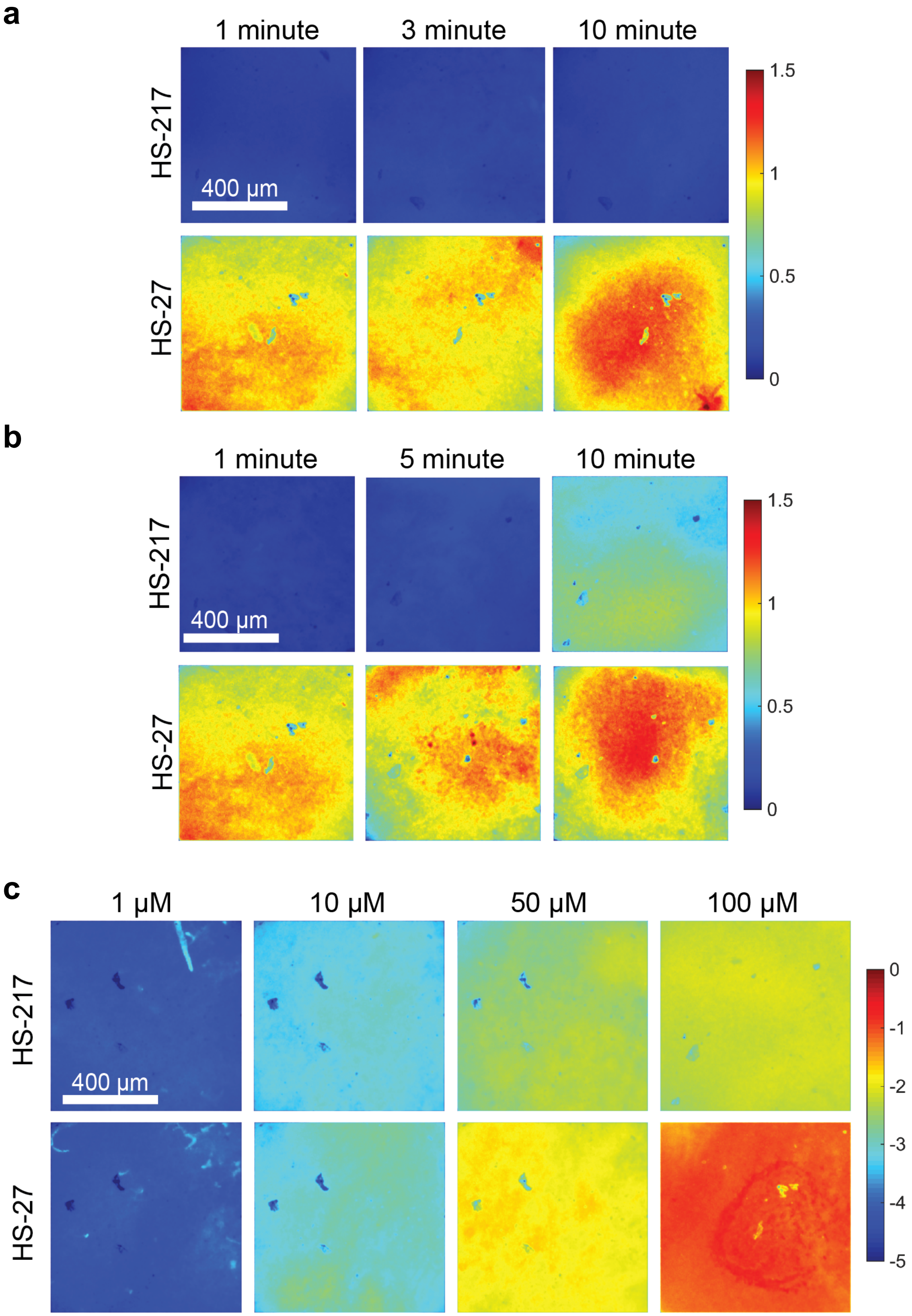

Supplementary Figure S1: Fluorescence images for *ex vivo* imaging optimization. (A) Representative images of biopsies taken from 4T1 tumors treated with 100 µM HS-27 for 1-minute either 1, 3, or 10-minutes post tissue excision. (B) Representative images of biopsies taken from 4T1 tumors treated with 100 µM HS-27

1-minute after tissue excision for either 1, 5, or 10-minutes. (C) Representative images of biopsies taken from 4T1 tumors treated 1-minute post tissue excision for 1-minute with either 1 μM, 10 μM, 50 μM, or 100 μM HS-27. Note images in panel (C) are shown on a log scale.

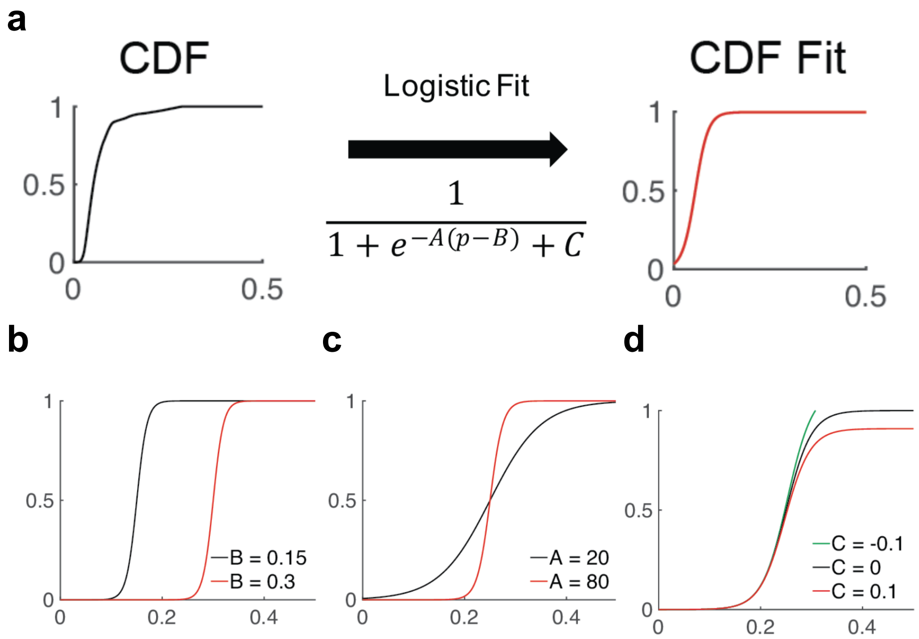

Supplementary Figure S2: Fitting a CDF to a logistic curve using three summary variables *A*, *B*, and *C*. (A) Each CDF was fit to a logistic curve of the form shown by minimizing the mean squared error between the fit curve and the true CDF. *A*, *B*, and *C* are the fit parameters and *p* is the pixel value. (B) The *B* parameter controls the left/right shift the CDF, with larger *B* values indicating a more right-shifted CDF. This reflects the mean fluorescence in the image. (C) The *A* parameter controls the slope of the CDF, with larger *A* values indicating a steeper slope. This reflects the overall variance within the image. (D) The *C* parameter controls how rapidly the slope changes at the top of the CDF. This reports on the mean and variance of the highest pixel values.
